# Integration of clinical and proteomic risk factors enhances prognostic modelling of incident vascular complications in type 2 diabetes

**DOI:** 10.1186/s12933-026-03083-6

**Published:** 2026-01-20

**Authors:** Yue Huang, Mauro Tutino, Archit Singh, Nigel William Rayner, Andrei Barysenka, Ozvan Bocher, Eleftheria Zeggini

**Affiliations:** 1 TUM School of Medicine and Health, Graduate School of Experimental Medicine, Technical University of Munich (TUM), 81675 Munich, Germany; 2 Institute of Translational Genomics, Helmholtz Zentrum München–German Research Center for Environmental Health, 85764 Neuherberg, Germany; 3 Munich School for Data Science (MUDS), Helmholtz Zentrum München–German Research Center for Environmental Health, 85764 Neuherberg, Germany; 4 Université Brest, Inserm, EFS, UMR 1078, GGB, F-29200 Brest, France; 5 TUM School of Medicine and Health, Technical University of Munich (TUM), TUM University Hospital, 81675 Munich, Germany

**Keywords:** Macrovascular/Microvascular complications, Type 2 diabetes, Proteomics, Cohort study, Long-term prognosis, Cross-ancestry generalizability, Model fairness

## Abstract

**Background:**

Type 2 diabetes complications manifest across various organs, but are fundamentally rooted in vascular dysfunction. This study aims to identify plasma protein signatures that improve prediction of macrovascular and microvascular complications in type 2 diabetes over classical clinical factors, assess the stability of their prognostic performance over time, and explore the cross-ancestry generalizability of the developed models.

**Methods:**

We analysed 2,923 plasma proteins in 917 European-ancestry UK Biobank participants with prevalent type 2 diabetes but no prior vascular disease at baseline. The primary outcomes were time to first macrovascular or microvascular complication, identified through ICD-10 codes during a mean follow-up of 10.41 years. Protein selection was performed using clinical-variables-prioritized LASSO Cox regression across 100 resamples to identify proteins offering predictive value beyond established clinical markers. Stably selected proteins were then integrated with and evaluated against clinical-only models using optimism-corrected C-index, time-dependent AUC and Brier score. We also conducted exploratory analyses to assess model generalizability in 116 European genetic outliers and in 80 Asian and 54 African ancestry participants within the UK Biobank.

**Results:**

For macrovascular outcomes, 37 proteins were selected, led by LRRC37A2, NT-proBNP, CHGA, APOD and STAB2. For microvascular complications, 9 proteins were selected, led by IL15, FAM3C and TNFSF11, with overall more moderate stability across resampling. The proteomics-integrated models significantly improved prediction of type 2 diabetes vascular complications beyond clinical markers (Harrell’s C: macrovascular 0.72 vs. 0.60; microvascular 0.67 vs. 0.62) and demonstrated stable prognostic accuracy over 10 years for macrovascular outcomes. In exploratory generalizability analyses, predictive gains of proteomics integration were maintained in European genetic outliers but diminished in African and Asian participants.

**Conclusions:**

Integrating proteomics with clinical data enhances risk prediction of type 2 diabetes vascular complications, especially for macrovascular outcomes. However, less precise prediction for microvascular complications and preliminary evidence of limited cross-ancestry generalizability highlight the need to expand targeted biomarker panels and quantification in larger, more ancestry-diverse cohorts to ensure effective and equitable clinical implementation of proteomics.

**Supplementary Information:**

The online version contains supplementary material available at 10.1186/s12933-026-03083-6.

## Background

Type 2 diabetes is a major global health concern, with vascular complications as the main driver of mortality and morbidity [1]. Much of vascular complications heterogeneity stems from differences in angiostructures, namely larger vessels versus smaller capillaries, and their distinct responses to chronic hyperglycaemia [2]. Macrovascular complications, such as cardiovascular disease and stroke, are leading causes of death, while microvascular complications, including nephropathy, retinopathy, and neuropathy, severely impact quality of life, leading to blindness, kidney failure, and limb amputation [2].

Prognosis models play a central role in managing type 2 diabetes progression but are often designed around individual end-organs, such as the heart (cardiopathy), brain (encephalopathy), kidneys (nephropathy), eyes (retinopathy) and nerves (neuropathy). This organ-specific approach, shaped by medical subspecialties, has influenced the development of widely used prediction models, including UKPDS [3], WATCH-DM [4], and RECODe [5], which largely focus on single types of complications. However, prevention and treatment strategies based on isolated vascular complications are becoming insufficient for long-term type 2 diabetes management, as complications often co-occur [2, 6] and evolve beyond limited traditional categories, including emerging liver-related conditions such as MASLD and NASH [7–9]. A more holistic, multi-system prognosis model is needed to improve patient outcomes by integrating prevention and care across specialties with coordinated targeted screening and early intervention. This can support more efficient use of healthcare resources by addressing the shared risk factors of pathologically interconnected complications, rather than preventing each in isolation.

Recently, proteomics has shown great promise as early biomarkers of a wide spectrum of diseases [10–13], including type 2 diabetes complications [14–16]. However, the models based solely on omics data often show limited efficacy for predictive purposes when compared to classical clinical variables which are more cost-efficient and easily accessible [17–19]. Specifically, for type 2 diabetes patients, routine assessment of novel circulating biomarkers, including proteins, is not yet recommended for cardiovascular risk stratification due to gaps in the evidence to support incremental discriminative power over established risk factors [20].

Here, we used Olink proteomics profiles from the UK Biobank [21] to integrate clinical and proteomics data to develop a unified predictive framework for the early screening of vascular complications in type 2 diabetes. We developed separate predictive models for macrovascular and microvascular outcomes to capture their distinct pathological processes. To identify proteins with independent and clinically meaningful contributions, we performed feature selection conditioned on established clinical variables. This strategy enables the integration of minimal protein signatures into existing clinical data, providing proof-of-concept for developing small, clinically translatable protein panels for vascular complication screening in type 2 diabetes patients.

## Methods

### Study participants and outcomes identification

Data were extracted from the UK Biobank (UKB), a large-scale, observational, long-term prospective biomedical cohort database in the UK [22]. A subset of these participants underwent plasma proteomic profiling through the UK Biobank Pharma Proteomics Project (UKB-PPP), which was largely randomly selected from the full UKB population [21].

Participants were classified as having prevalent type 2 diabetes at baseline based on two criteria [23]: (1) if their first-occurrence date for type 2 diabetes (Data-Field 130708) was before the baseline assessment date (Data-Field 53), or (2) if they were diagnosed after the baseline date but had glycated haemoglobin (HbA1c) levels (Data-Field 30750) exceeding 48 mmol/mol at the time of the baseline measurement. The first-occurrence date was determined using an integrated approach that combined data from self-reports, general practice records, electronic health records (EHR) linked to the National Health Service, and the death registry.

Among these prevalent type 2 diabetes participants (*n* = 21,139), only a subset had full-panel proteomic profiling available as part of the UKB-PPP. Baseline characteristics of participants with and without full proteomic data were broadly similar (Table S1), supporting representativeness of the analysed subset. We further selected participants of European ancestry with prevalent type 2 diabetes but no prior history of vascular disease at baseline measurement (blood sample collection) as the main dataset for prognosis model development (2006–2010; *n* = 917). The study participant selection flow is detailed in Figure S1.

The primary model was trained on participants of European ancestry, namely, those who self-identified as ‘White British’ in the questionnaire and were previously reported of European ancestry based on the genetic principal component analysis (Data-Field 22006). This decision was based on substantial evidence [24, 25] that the progression to vascular complications in diabetes differs among global populations, supporting the need for ancestry-specific models. This was confirmed in the UKB where we observed heterogeneity in temporal risk patterns for microvascular and macrovascular complications between ancestry groups (Figure S2). Ancestry classification was based on self-reported data, grouped into five major categories following the 2021 Census of England and Wales: European, Asian, African, Mixed, and Other. Participants of Asian (*n* = 80) and African (*n* = 54) ancestry, as well as European genetic outliers (*n* = 116) who passed all the selection criteria, were used as test sets to explore models’ cross-ancestry generalizability (see section Cross-ancestry Generalizability Analysis).

Due to distinct pathophysiology [2] and risk profiles (Figure S2), we modelled macrovascular and microvascular complications separately. The primary outcomes were defined as the time-to-first macrovascular or microvascular complication, measured in years since baseline, based on the earliest ICD-10 codes for each category [26–28] (Table S2). Notably, our outcome definitions also included complications often omitted in prior studies, like liver-related vascular and metabolic conditions such as hepatic fibrosis and steatosis. These conditions are gaining increasing attention as microvascular complications of type 2 diabetes, with recent consensus reports calling for systematic screening and greater clinical awareness [8, 9, 29]. Individuals with any pre-existing complications at baseline were excluded. Additionally, given sample size constraints, we treated two complication types as independent events without accounting for potential interplay - such as onset of one complication type accelerating the onset of the other. Participants who developed both complication type during follow-up were included as incident cases in both models, with potentially different onset times for each. Censoring dates were defined as the earliest of following dates: hospital or death record updates, loss to follow-up (participants who left the UK or withdrew consent), death, or December 31, 2019, to avoid biases caused by disruptions in healthcare access and mortality patterns during the COVID-19 pandemic.

### Proteomic data and clinical variable processing

Proteomic was profiled on plasma samples from 54,219 individuals in UKB with the Olink Explore 3072 platform (2,923 proteins). Details regarding the collection, study design, and preprocessing steps can be found in the UK Biobank Pharma Proteomics Project (UKB-PPP) [12, 21]. Protein quality control (QC) was conducted on the entire UKB-PPP cohort. Protein assays with over 40% of samples below the limit of detection (LOD) were excluded. For remaining assays, values below the LOD were retained as actual values, as recommended by Olink supplier. To further improve data reliability, samples and proteins with more than 20% missing values were sequentially excluded. The rest of missing protein values were imputed using k-nearest neighbour imputation. Outliers were handled through one-sided winsorizing at the 99th percentile to minimize their influence. To mitigate confounding, both batch effects and biological covariates were adjusted for using linear mixed model prior to training the model. Technical covariates, including assessment centres, sample run plates, and sample well, were adjusted as random effects, while biological covariates such as age, sex, BMI, season of sample collection, and fasting time were adjusted as fixed effects. A total of 2,130 proteins passed QC, and the residuals from this adjustment were used as the post-QCed values in the modelling procedure.

Clinical predictor variables for microvascular and macrovascular outcomes were selected by aggregating biomarkers from existing risk engines [3–5, 30] for diabetes complications. A single set of clinical variables was used for both macrovascular and microvascular outcomes, ensuring consistency, reducing model complexity, and enhancing comparability across the two complication types. The final clinical biomarker set included age, sex, HbA1c, glucose, BMI, waist-to-hip ratio, total cholesterol, HDL, LDL, triglycerides, alanine aminotransferase, creatinine, eGFR, systolic and diastolic blood pressure (SBP and DBP), and pulse rate. Two versions of eGFR were calculated using cystatin C and creatinine with the EKFC eGFRcys [31] equation and the EKFC eGFRcr [32] equation via the *nephron* (v1.4) [33] R package. Missing clinical variables were imputed using the *mice* (v3.17.0) [34] R package.

### Robust protein variable selection and model development

The overall modelling workflow is summarized in Figure S3 . Protein selection was performed using least absolute shrinkage and selection operator (LASSO) Cox regression with customized penalty scheme ‘favouring’ clinical variables [35, 36]. More precisely, we impose a penalty only to the proteomics variables, leaving the clinical ones unpenalized by assigning their penalty weights to zero, serving as a foundational framework for identifying proteins with additional prognostic value over clinical variables. On the other hand, integrating extensive clinical information can lead to a smaller set of omics predictors being selected [19], which facilitates a more robust yet sparse set of candidate biomarkers.

Additionally, LASSO feature selection is known to exhibit instability, particularly in omics studies with high-dimensional data (small n/p ratios). To address this, we adopted a consensus-based nested resampling approach [37], implementing 100 repeated subsampling iterations with an 80/20 train-test split stratified by outcome status. Within each iteration, nested five-fold cross-validation on the training set was used to optimize hyperparameter (λ), selecting the protein set that achieved the highest prediction performance (based on the c-index) in the internal test set. Protein selection stability was quantified as the proportion of times each protein was selected across 100 resampling runs to derive a consensus feature set. To determine an appropriate inclusion threshold for defining stable predictors, we first estimated the maximum number of parameters that could be reliably supported by our sample size, given the observed event rates and anticipated model discrimination [38]. Based on these calculations, approximately 30–40 parameters could be estimated for the macrovascular model and 20–30 for the microvascular model (Table S3). Guided by these limits, we set the inclusion threshold at ≥ 20% across resampling iterations to balance model stability with feasible parameter estimation, while avoiding overly stringent selection that could exclude potentially informative proteins. Proteins meeting this ≥ 20% inclusion threshold were then combined with pre-defined clinical variables (age, sex, and 15 established biomarkers) to form proteomics-integrated models, which served as the primary framework for all subsequent analyses and reporting.

To explore the potential for more parsimonious models, we additionally conducted sensitivity analyses by (1) applying a stricter selection threshold (≥ 30%) and (2) performing backward elimination based on the Akaike Information Criterion (AIC) on the protein subset derived from the 20% threshold, while retaining all clinical variables.

### Model internal evaluation and interpretation

The final protein-integrated model, along with alternative versions derived from sensitivity analyses, were then internally evaluated against clinical-only models using 100 bootstrap resamples (sampling with replacement) of the original dataset for optimism correction, as it provides more efficient and less biased estimates of model performance, particularly in moderately sized datasets [39, 40]. Performance metrics included Harrell’s C-index, Uno’s C-index, time-dependent AUC, and Brier score. All metrics were averaged across the 100 bootstrap resamples to obtain optimism-corrected estimates. The Brier score measures prediction accuracy as the mean squared difference between the predicted probability of experiencing event at a specific time and the actual outcome (event = 1, no event = 0), with lower scores reflect greater accuracy. For reference, null Brier scores were calculated using the overall incidence rates of macrovascular and microvascular complications estimated in this population. This represents a non-informative model that assign every individual the population-level complication probability, serving as baseline benchmarks for model performance. For all protein predictors in the final model, missing value imputation rates were reported to assess completeness and reliability of original data, and variance inflation factors (VIF) were computed to evaluate multicollinearity among predictors.

To illustrate the relative effects of each predictor within the final multivariable model, hazard ratios (per 1-SD increase) with 95% confidence intervals were reported. In addition, we quantified the temporal predictive importance of each selected protein through time-dependent permutation-based feature importance analysis, measured as the change in Brier score after permuting individual predictors to reflect each protein’s unique contribution to overall predictive accuracy across the follow-up period [41].

### Generalizability analysis across ancestry and genetic subgroups

To assess the potential generalizability and fairness of the developed models across ancestries, we first estimated the statistical power required for external validation. As there is currently no closed-form solution for calculating power or minimum sample size for external validation of time-to-event prediction models [42], we approximated the sample size requirement for a logistic regression on 10-year binary outcomes [43]. Given the observed 10-year incidence rates and model discrimination (C-index 0.75 for macrovascular and 0.70 for microvascular outcomes), the minimum sample sizes for adequately powered external validation were approximated at 476 and 471, respectively.

As the non-European cohorts were smaller than these thresholds, subsequent analyses were considered exploratory. Both the clinical-only and clinical + proteomics models were trained in genetically homogeneous European participants (European baseline) defined by the UK Biobank genetic ethnic grouping (Data-Field 22006), which is based on principal component analysis of genome-wide genotyping data [44]. These models were then applied to European genetic outliers (*n* = 116), representing a genetically more heterogeneous subset of self-reported Europeans outside the genetically homogeneous set. For broader ancestry evaluation, the same models were additionally applied to Asian (*n* = 80) and African (*n* = 54) ancestry test sets. Pairwise comparisons of the clinical-only and clinical + proteomics models within each ancestry group were performed to assess whether the incremental performance from proteomic integration persisted beyond the European training population. This design allowed evaluation of the incremental contribution of proteomics within each ancestry group, isolating ancestry effects without conflating them with sample size or baseline risk profile differences. Model performance metrics were reported using optimism-corrected bootstrap estimates for European baseline cohort and direct out-of-sample estimates for other subgroups. In addition, ancestry-specific performance gains from proteomics integration using models trained in European baseline participants (Δ = clinical + proteomics vs. clinical) were estimated using paired performance metrics across 1,000 bootstrap resamples (sampling with replacement) within each ancestry group and summarised as mean (95% CI). Due to limited sample sizes in non-European groups, only overall C-index and integrated Brier score are reported, as time-dependent metrics could not be reliably estimated with small sample sizes.

All statistical analysis was performed in R (v4.4.1). Main model development and validation was with *mlr3* (v0.22.1) [45] and *mlr3proba* (v0.7.1) [46] packages. Power calculations were performed with *pmsampsize* (v1.1.3) [38] for model development and *pmvalsampsize* (v0.1.0) [43] for external validation.

This study adheres to the TRIPOD + AI [33] reporting guidelines for the development and validation of a prediction model, as documented in the Supplementary Material.

### Biomarker database search

To assess the relevance and novelty of the selected candidate biomarkers, we searched for their prior biomarker designation, including associations with diagnostic, prognostic, or therapeutic applications in the *MarkerDB 2.0* [47] (accessed on February 15th 2025). Each protein was queried using its official gene symbol, UniProt ID, and alternative names where applicable.

## Results

The baseline characteristics of the study participants, the mean follow-up time and event counts, stratified by ancestry, are summarized in Table S4, and corresponding Kaplan–Meier survival curves are shown in Figure S2. Among 917 type 2 diabetes patients of European ancestry, 241 developed macrovascular and 297 developed microvascular complications, with 126 individuals overlapping between the two groups (i.e., developing both types), over a mean follow-up of 10.69 years. (Table 1) The distribution of first-incident events by organ system is shown in Figure S4, illustrating that macrovascular outcomes were dominated by cardiac events, whereas microvascular outcomes were most frequently driven by kidney disease, followed by retinopathy. A substantial overlap was observed across affected organ systems, underscoring the interrelated nature of vascular complications and supporting the need for a coordinated, pan-screening modelling framework.

**Table 1:** Baseline characteristics of participants with prevalent type 2 diabetes of European ancestry in the UK biobank (*n* = 917) at baseline, stratified by incident macrovascular and microvascular complications during follow-up, shown separately for each outcome category

Participants with/ without incident event of	Macrovascular Complications		p	Microvascular Complications		p
	0	1		0	1	
n	676	241^a^		620	297^b^	
Age at baseline measurement (year)	59.85 (6.66)	62.12 (5.94)	< 0.001	59.63 (6.68)	62.15 (5.94)	< 0.001
Age at T2D diagnosed (year)	57.63 (7.43)	60.12 (6.43)	< 0.001	57.51 (7.37)	59.91 (6.75)	< 0.001
T2D duration at baseline measurement (year)	2.71 (3.94)	2.50 (3.49)	0.449	2.61 (3.85)	2.75 (3.77)	0.622
Sex = Male (%)	402 (59.5)	167 (69.3)	0.009	380 (61.3)	189 (63.6)	0.54
HbA1c (mmol/mol)	54.56 (13.58)	56.73 (15.54)	0.043	54.31 (13.32)	56.84 (15.62)	0.012
Glucose (mmol/L)	7.98 (3.42)	8.00 (3.41)	0.94	7.75 (3.22)	8.50 (3.77)	0.003
Pulse rate (bpm)	75.82 (13.34)	77.02 (13.66)	0.233	75.98 (13.27)	76.47 (13.77)	0.61
SBP (mmHg)	143.92 (17.00)	144.95 (17.33)	0.422	143.99 (16.93)	144.61 (17.43)	0.61
DBP (mmHg)	84.12 (10.11)	82.97 (10.25)	0.13	84.54 (9.99)	82.30 (10.34)	0.002
BMI (kg/m^2^)	32.33 (5.87)	32.24 (5.55)	0.839	32.27 (5.75)	32.38 (5.87)	0.775
Waist-to-hip ratio	0.95 (0.08)	0.96 (0.08)	0.024	0.95 (0.08)	0.96 (0.08)	0.151
Cholesterol (mmol/L)	4.74 (1.21)	4.66 (1.21)	0.382	4.77 (1.20)	4.63 (1.23)	0.131
HDL cholesterol (mmol/L)	1.18 (0.29)	1.15 (0.29)	0.239	1.18 (0.28)	1.15 (0.29)	0.13
LDL cholesterol (mmol/L)	2.88 (0.90)	2.85 (0.91)	0.642	2.90 (0.91)	2.82 (0.91)	0.274
Triglycerides (mmol/L)	2.40 (1.34)	2.31 (1.31)	0.356	2.39 (1.38)	2.35 (1.23)	0.691
Creatinine (umol/L)	72.33 (16.45)	75.09 (19.13)	0.037	71.56 (15.40)	76.22 (20.22)	< 0.001
eGFR(EKFC eGFRcr) (mL/min/1.73 m^2^)	0.58 (0.14)	0.56 (0.14)	0.084	0.59 (0.13)	0.55 (0.16)	< 0.001
eGFR(EKFC eGFRcys) (mL/min/1.73 m^2^)	80.12 (12.99)	76.23 (13.41)	< 0.001	81.16 (12.10)	74.77 (14.36)	< 0.001
Alanine aminotransferase (IU/L)	33.39 (21.20)	30.02 (18.69)	0.033	33.41 (20.65)	30.63 (20.47)	0.063
Albumin (g/L)	45.15 (2.80)	44.88 (2.92)	0.243	45.16 (2.83)	44.91 (2.84)	0.237
Follow-up time (year)	10.55 (1.73)	10.00 (2.24)	< 0.001	10.47 (1.87)	10.28 (1.94)	0.168
Onset time of corresponding complication (year since baseline)	NA	5.63 (3.14)	NA	NA	6.21 (3.07)	NA

Continuous variables are presented as mean(SD), categorical variables are presented as n (%). P-values for comparisons across ancestry subgroups were calculated using t-tests (for continuous variables) or chi-squared tests (for categorical variables)

a, b 126 participants developed both complication types and are counted in both the macrovascular (*n* = 241) and microvascular (*n* = 297) groups

### Robust and strongly associated prognostic proteins of vascular complications

Across 100 repeated clinical-prioritized LASSO-Cox iterations, substantial variability was observed in both feature selection and intermediate model test-set performance (Table S5), as expected in high-dimensional settings. The mean (± SD) number of proteins selected per iteration was 26.3 (± 13.5) for macrovascular and 7.03 (± 8.8) for microvascular models, in addition to the 17 predefined clinical variables included in every model. These observations motivated the use of an aggregated, consensus-based feature-selection strategy to identify proteins with consistent prognostic relevance across resampling iterations. Based on selection in more than 20% of iterations criteria, we identified 37 proteins predictive of macrovascular and 9 of microvascular complications, conditioned on established clinical variables (Table S6). All selected proteins had low proportions of imputed values (maximum 6.9%; Table S7) indicating high data completeness. Inter-protein correlations among the selected proteins were generally low, with 91.6% of pairwise correlations having |r| < 0.2 (highest between GAST and CHGA within macrovascular outcomes, *r* = 0.74; Figure S5), indicating low redundancy among predictors. This was further supported by low variance inflation factors (VIFs) from the final multivariable Cox models (Table S8), confirm minimal multicollinearity among predictors.

**Fig. 1:**
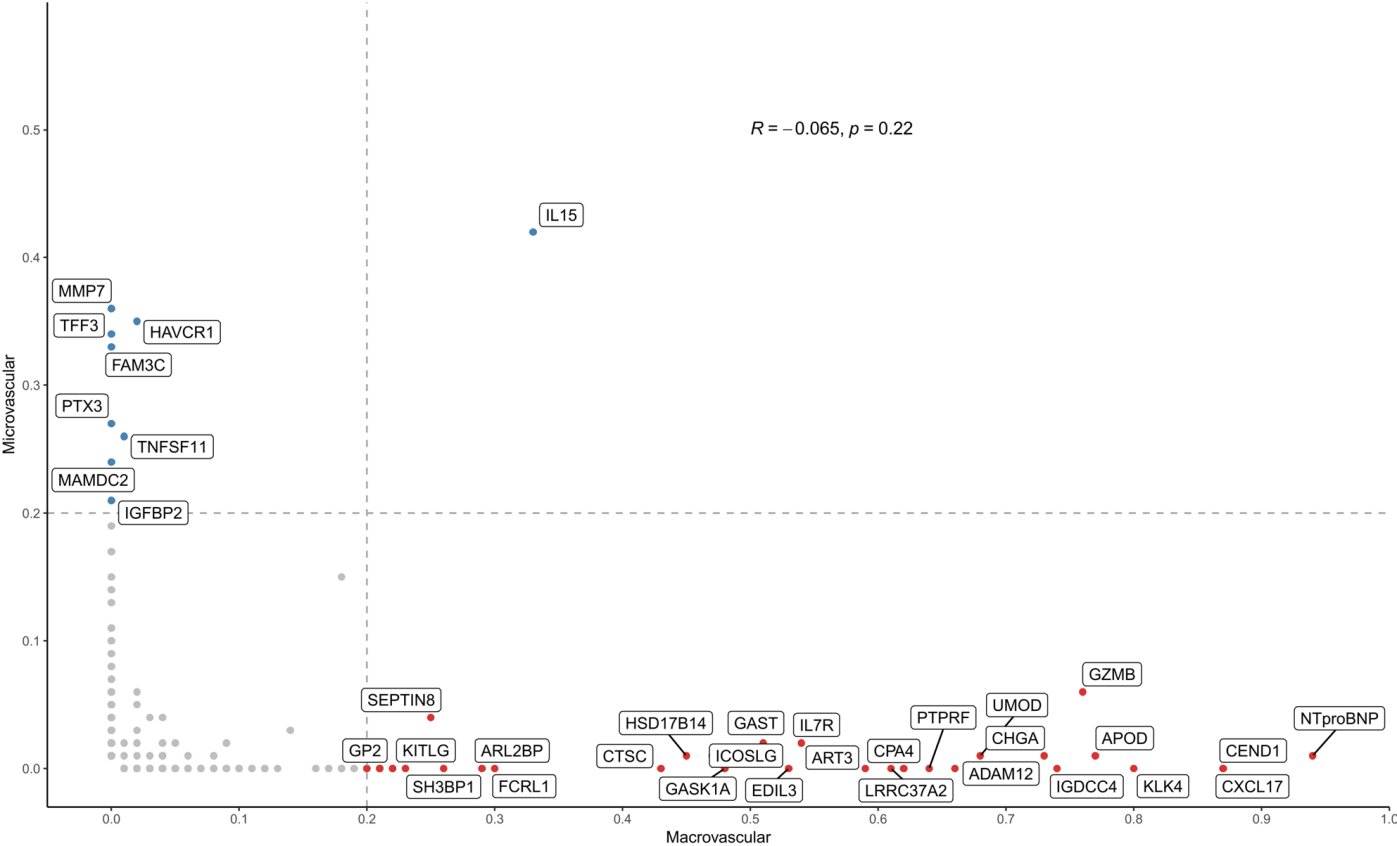
Protein selection frequency conditioned on clinical variables in LASSO-Cox models for macrovascular (x-axis) and microvascular (y-axis) complications. Frequencies reflect feature selection stability across 100 stratified 80/20 train-test splits. Proteins selected in ≥ 20% of iterations are labelled, those more frequently selected for macrovascular than microvascular outcomes are shown in red, and vice versa for microvascular outcomes in blue

Corresponding pairwise co-selection patterns (Figure S6) further revealed clusters of proteins frequently co-selected across resampling iterations, reflecting coordinated and complementary contributions to the overall prognosis within each outcome. In contrast, protein selection frequencies showed low concordance between macrovascular and microvascular complications (*R* = -0.065, *P* = 0.22; Fig. 1). One exception was IL-15, the only selected protein shared between macrovascular and microvascular models with a selection frequency of 33% and 42%, respectively.

Out of the 37 proteins selected for macrovascular complications, NT-proBNP was the most consistently informative biomarker, selected in 94% of resampling iterations. Other highly robust predictors included CEND1, CXCL17 and KLK4 (selected in > 80% of iterations), followed by APOD, GZMB, IGDCC4, and CHGA (70–80%). UMOD, ADAM12, PTPRF, CPA4 and LRRC37A2 were selected in 60–70% of iterations, and the remaining 24 proteins were selected in less than 60% of iterations. Out of the 9 proteins selected for microvascular complications, no protein demonstrated highly robust predictive value, with IL-15 as the most frequently selected protein at 42% frequency. Other proteins include MMP7, HAVCR1, TFF3, FAM3C, PTX3, TNFSF11, MAMDC2 and IGFBP2.

**Fig. 2:**
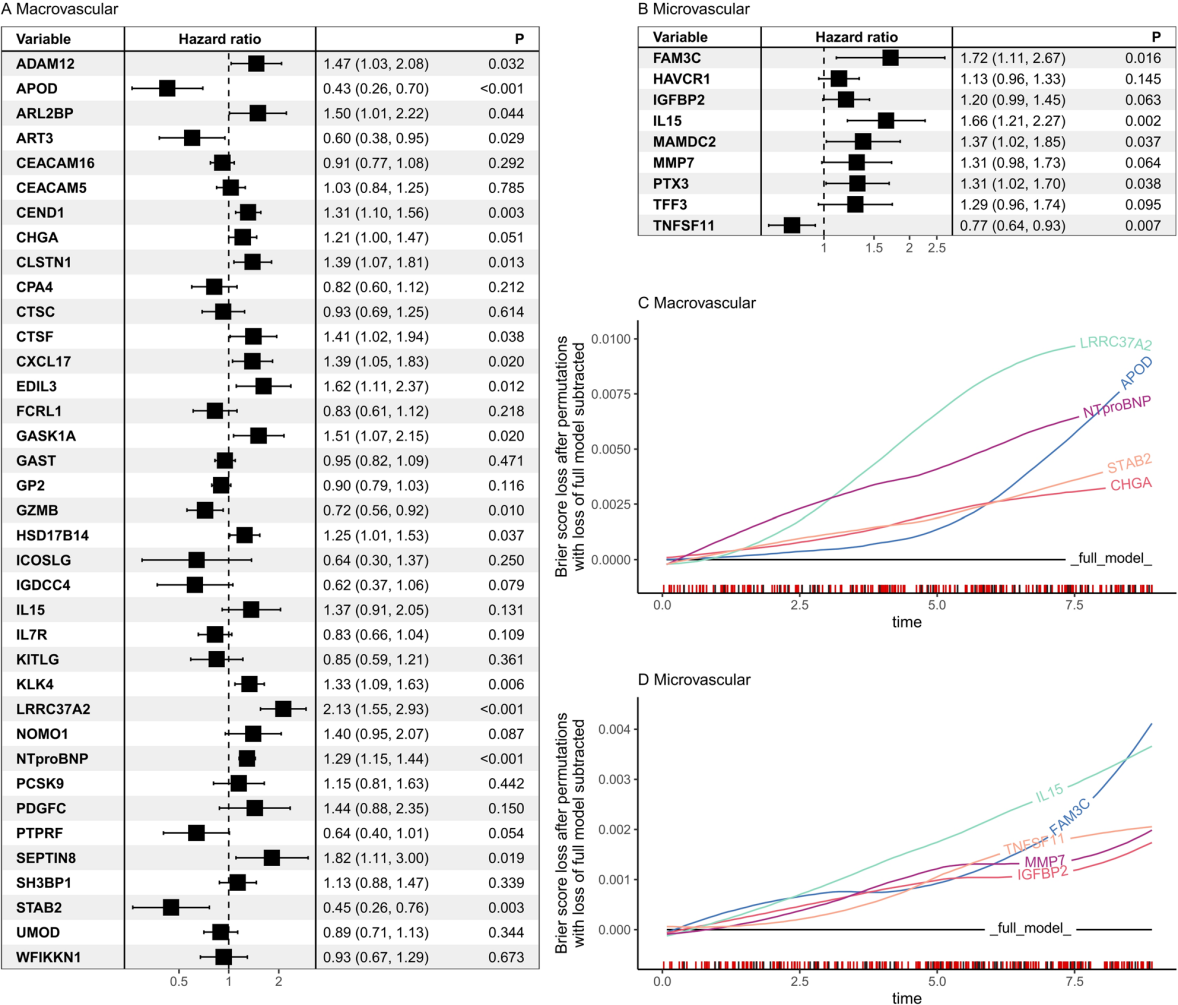
Effect sizes and time-dependent predictive importance of selected proteins in the final clinical + proteomics models for vascular complications. Forest plots show adjusted hazard ratios (HRs) per standard deviation (SD) increase in post-QCed protein level, each mutually adjusted for all other covariates including other proteins in the multivariable model, with 95% confidence intervals (CIs) and p-values for macrovascular (**A**) and microvascular (**B**) complications. Clinical covariates were included in the models but are not displayed. Panels (**C**) and (**D**) present time-dependent permutation-based feature importance for macrovascular and microvascular models, respectively, quantified as the change in prediction error (Brier score) after permuting each variable; only the top five protein predictors are shown

While feature selection frequency reflects the robustness and reproducibility of each protein across resampled datasets, the effect sizes from the final multivariable model, expressed as hazard ratios per 1-SD increase, quantify the magnitude and direction of their relative associations with complications (Fig. 2A–B; detailed in Table S8). In the macrovascular model, LRRC37A2 (HR 1.417, 95% CI [1.224–1.64], *P* < 0.001), NTproBNP (HR 1.375 [1.195–1.582], *P* < 0.001) were the proteins most strongly associated with increased risk. Conversely, APOD (HR 0.684 [0.549, 0.852], *P* = 0.001) and STAB2 (HR 0.785 [0.668–0.922], *P* = 0.003) were significantly associated with lower risk. For the microvascular model, IL15 (HR 1.209 [1.074–1.36], *P* = 0.02) and FAM3C (HR 1.205 [1.035–1.402], *P* = 0.016) exhibited the strongest associations with increased complication risk. In contrast, TNFSF11 (HR 0.843 [0.744–0.955], *P* = 0.007) was the only protein significantly associated with decreased risk of microvascular outcomes.

In addition to effect sizes, we also provided a dynamic view of each protein’s prognostic contribution over the follow-up period. Figure 2C and D show the top five proteins ranked by time-dependent permutation-based feature importance. LRRC37A2, NT-proBNP, APOD, STAB2, and KLK4 emerged as the most influential proteins in the macrovascular model, while FAM3C, IL15, TNFSF11, MMP7, and IGFBP2 ranked highest for microvascular outcomes.

Comparison with biomarker databases confirmed that several selected protein predictors were previously established biomarkers (Table S9). NT-proBNP is a diagnostic marker for congestive heart failure, HAVCR1 (also known as TIM-1/KIM-1) for acute kidney injury, and CHGA for bone metastases. Additionally, GAST and CHGA are both diagnostic biomarkers for enterochromaffin-like cell hyperplasia.

### Proteomics improves risk stratification and individualized follow-up scheduling

The main reporting clinical + proteomics model was based on clinical variables combined with proteins retained in more than 20% of resampling iterations. A sensitivity analysis using two more parsimonious models, one retaining proteins with a ≥ 30% selection frequency threshold and another derived from backward selection on the ≥ 20% preselected set, yielded comparable performance (Table S10). For macrovascular complications, backward selection removed 11 proteins without compromising predictive accuracy (C-index improved by 0.01), indicating that the core predictive signal could be further concentrated within a subset of proteins, whereas for microvascular complications, no proteins could be further excluded. Nonetheless, the full ≥ 20% model was retained for downstream analyses to capture a broader range of potentially informative signals in current exploratory phase.

**Fig. 3:**
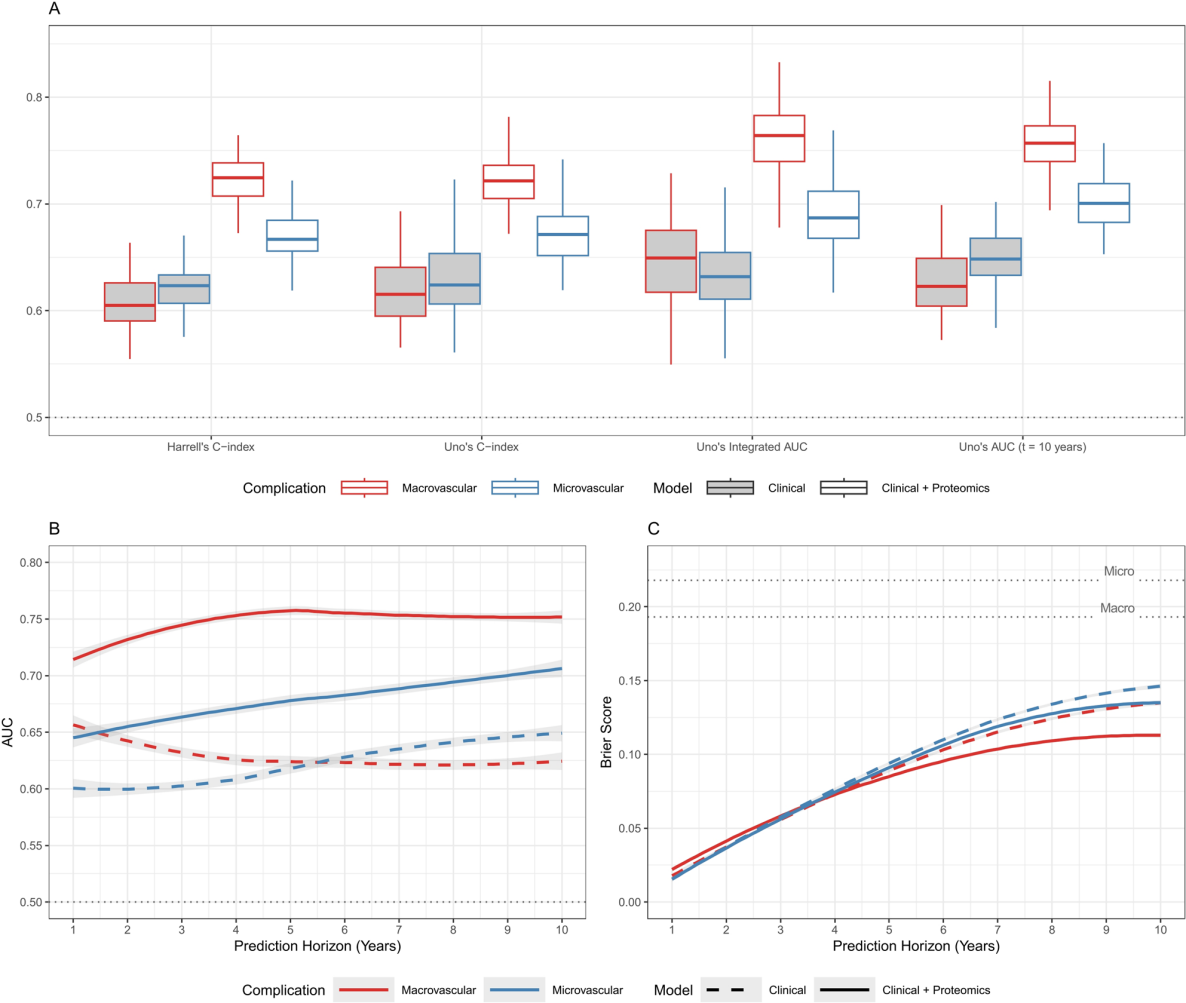
Prediction performance benchmarking of clinical models vs. clinical + proteomics models for vascular complication risk prediction in T2D patients of European ancestry. **A** Overall Discrimination measures. **B** Time-dependent AUC. **C** Time-dependent Brier score (dotted lines indicate the null Brier score, i.e., the expected Brier score when every individual is assigned the average event rate in the population for micro- or macrovascular complications, respectively)

The integration of proteomics data consistently improved predictive performance over clinical risk factors across all evaluated metrics for both macrovascular and microvascular complications (Fig. 3). Models incorporating proteomics data demonstrated superior performance in ranking patients by relative risk, as reflected in higher median values and lower variability of Harrell’s C-index, Uno’s C-index, Uno’s Integrated AUC, and Uno’s AUC at 10 years (Fig. 3A). These metrics quantify how well the model differentiates and ranks individuals who will experience complications from who will not, potentially guiding clinical resource distribution by prioritizing high-risk patients for early intervention.

While single-time metrics provide a broad assessment of predictive power, time-dependent AUC tracks how well the model distinguishes between high- and low-risk individuals over time across the prediction horizon (Fig. 3B). For macrovascular complications, the proteomics-integrated model generally demonstrated high predictive accuracy (AUC > 0.75) during the whole follow-up period. In contrast, the clinical-only model showed a much earlier and steeper decline in the effectiveness of prediction. For microvascular complications, adding proteomics improved short-term discrimination from near-random levels (AUC ~ 0.55) to a more meaningful level (AUC ~ 0.65), though the improvement in general was less pronounced than that observed for macrovascular outcomes. Over time, the performance of the proteomics-integrated model continued to improve, suggesting that while patients at higher long-term risk can be identified, the exact timing of microvascular events is less predictable.

Beyond ranking patients by risk, time-dependent Brier scores assess the accuracy of absolute risk estimates, which is crucial for individualized prognosis. As baseline benchmarks for model performance, the null Brier scores were 0.193 for macrovascular and 0.218 for microvascular complications, based on the corresponding incidence rates of 26.1% and 32.2% in this population. These reflect expected values from a non-informative model that assigns each individual the population-level complication probability. As shown in Fig. 3C, all four models demonstrated substantially lower Brier scores compared to a null Brier score (dotted line), supporting their added value for absolute risk estimation across all prediction horizons. Additionally, the proteomics-integrated model consistently achieved lower Brier scores throughout the prediction horizon for both complication types, particularly for macrovascular complications at later time points, indicating superior accuracy in absolute risk estimation.

### Ancestry-dependent predictive gains of proteomics integration

A key question in proteomics-integrated prognosis is whether it can provide ancestry-agnostic predictive improvement to guarantee equitable access to global preventative health care. As the non-European cohorts were underpowered for formal external validation of the developed model, we instead performed exploratory pairwise comparisons of the proteomics-integrated versus clinical-only models within each ancestry group. Both the clinical-only and clinical + proteomics models were trained in European participants. This design isolates the incremental contribution of proteomics without conflating it with population-specific baseline risk profiles or sample size.

**Fig. 4:**
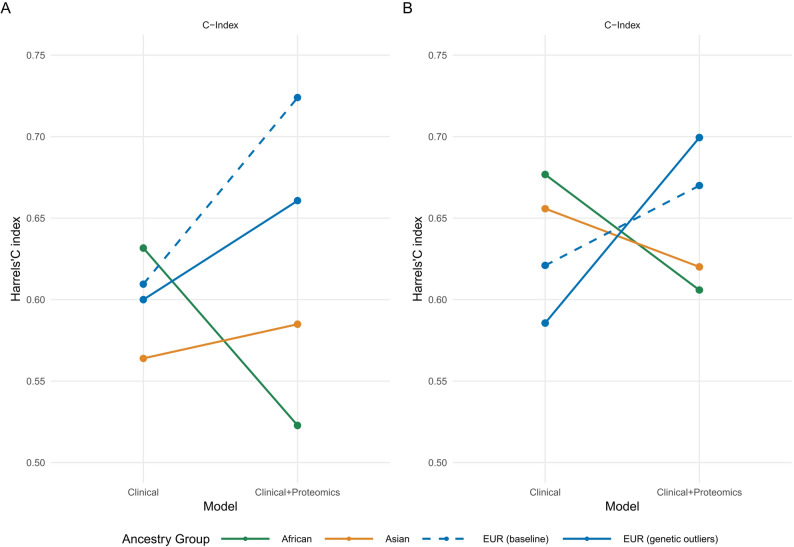
Cross-ancestry predictive performance of clinical-only versus. clinical + proteomics models for vascular complications in type 2 diabetes. Performance is shown separately for macrovascular A and microvascular B outcomes. Models were trained on genetically similar European baseline participants and tested in three external groups (all excluded from model training): (1) European genetic outliers (n = 116), (2) African ancestry (n = 54), and (3) Asian ancestry (n = 80) participants, to assess trans-ancestry transferability. Harrell’s C-index is presented for all groups. Metrics for the European baseline training group are optimism-corrected using 100 bootstrap resamples, while metrics for European genetic outliers, African, and Asian participants are direct out-of-sample estimates

Across ancestry groups, the incremental predictive value of proteomics integration showed a clear dependence on genetic distance from the European training population (Fig. 4; detailed in Table S11). In the European baseline cohort, proteomics integration yielded a substantial improvement in discrimination for macrovascular outcomes (Δ C-index = 0.125, 95% CI [0.072, 0.186]) and more modest improvements for microvascular outcomes (Δ C-index = 0.043 [-0.018–0.102]).

In European genetic outliers, absolute performance was attenuated, but the clinical + proteomics model retained a positive mean predictive gain but increased uncertainty for macrovascular outcomes (Δ C-index = 0.060 [− 0.057, 0.167]) and for microvascular outcomes (Δ C-index = 0.114 [0.040, 0.188]), as shown by modest upward slopes, supporting robustness of proteomic integration within a more genetically heterogeneous European subset.

In contrast, proteomics-related performance gains were diminished or reversed in Asian- and African-ancestry groups, as reflected by flattened or downward slopes. In these populations, mean bootstrap estimates of Δ C-index were small and even negative, and in African-ancestry participants the mean effect was negative for both macrovascular and microvascular outcomes, providing preliminary evidence that generalizability and predictive gains of protein-integrated model decrease with greater genetic distance from the discovery population.

## Discussion

Here, we present a proof of concept for a strategy to develop small protein panels that complement clinical risk stratification and are potentially feasible for translation into clinical settings for vascular complication screening in type 2 diabetes. Our study comprehensively included traditional macrovascular and microvascular complications, as well as emerging liver-related vascular and metabolic conditions increasingly recognized in type 2 diabetes. We grouped complications based on shared vascular pathology - macrovascular and microvascular - rather than by end-organ, aligning with the ultimate goal of preventive or therapeutic strategies, in which drugs typically circulate systemically and impact the whole vascular systems rather than specific organs [6]. Nevertheless, there has been ongoing debate regarding the classification of certain conditions [48], such as liver disease [2, 49, 50], which displays features of both metabolic and microvascular dysfunction. From a practical standpoint, however, including such outcomes in prognostic evaluation and management remains potentially valuable, as it enables a more comprehensive assessment of diabetes-related complications and may ultimately inform strategies to improve patient outcomes. Additionally, our feature selection was grounded in current medical knowledge and clinical practice, incorporating a comprehensive set of established clinical predictors to ensure that each selected protein adds prognostic value rather than duplicating existing information. This approach minimizes redundancy caused by correlations among predictors and helps to ensures that the final protein panel offers distinct, clinically informative contributions.

Although predictive proteins are not necessarily causal, they may reflect early pathological processes. There has been a long-standing debate about whether microvascular and macrovascular complications in type 2 diabetes are distinct pathological entities or a continuum of vascular disease [51]. Our protein selection results with largely non-overlapping protein signatures support their pathological distinctiveness in early stages, with the exception of the inflammation pathway captured by IL-15 in both vascular types.

Comparison of our protein selection results with clinically approved biomarker databases supports the validity of our findings when known markers are identified, while also highlighting the novelty of proteins not previously reported. For macrovascular complications, NT-proBNP was reaffirmed as a strong independent prognostic marker, while LRRC37A2 emerged with novel prognostic potential with even larger partial effect sizes than NT-proBNP. Conversely, APOD and STAB2, with strong negative association and variable importance with risk, may inform macrovascular-targeted therapies. For microvascular outcomes, we detected HAVCR1 (KIM-1), a known biomarker for acute kidney injury, along with FAM3C and IL15, which indicated larger effect size and variable importance for further investigation. TNFSF11, the only protein negatively associated with microvascular risk, may represent a biologically protective signal which needs to be further validated. GAST and CHGA, which were associated with macrovascular outcomes, are also known biomarkers of gastrointestinal disorders, suggesting a potential biological intersection between digestive comorbidities and vascular complications in type 2 diabetes, an area that warrants further research.

Current clinical guidelines recommend uniform annual follow-up to screen for complications for type 2 diabetes patients [52], but this one-size-fits-all approach does not account for varying levels of risk and can lead to inefficiencies in care delivery. Our findings may complement these guidelines by offering a data-driven insight to personalize follow-up schedules based on predicted complication type and temporal model performance. Based on the overall high and stable performance of the proteomics-integrated model for macrovascular complications, follow-up intervals could potentially be adjusted without compromising prognostic reliability. In contrast, for microvascular complications, while patients at higher long-term risk can be identified, the exact timing of microvascular events is less predictable, which aligns with the typically more silent and gradual progression of microvascular complications [54]. Thus, more frequent and intensive monitoring, potentially supported by specialized imaging or further lab tests, remains crucial to compensate for the model’s limited ability to detect acute cases to maintain clinical vigilance. These results support a stratified follow-up approach, where monitoring frequency is adjusted based on predicted complication type to ensure patients receive timely and appropriately spaced reassessment and ultimately improve care efficiency.

This study has several limitations. First, the research was conducted within a single cohort, without external validation in independent studies. While bootstrap-based estimates provide internally optimism-corrected performance metrics, they do not constitute independent internal validation. Second, we modelled macrovascular and microvascular complications independently without considering the interplay of comorbidities. While advanced methods such as multistate models could address these complexities, their application requires larger sample sizes due to the increased number of parameters to estimate. Thirdly, our protein-based models are inherently limited by the current proteomic profiling technologies which are majorly based on relative quantification and do not report protein concentration in standardized units [55]. From the biological insight perspective, our analysis is based on circulating, blood-accessible and targeted proteomics, focusing on soluble proteins primarily located in vesicles and extracellular matrix compartments. While this approach aligns with clinical screening purposes, it does not capture the full spectrum of biological processes, particularly tissue-specific and intracellular mechanisms.

Despite these limitations, our study provides timely insights into how future improvements, particularly through the advancement of population-based proteomics profiling, can enhance the prediction of vascular complications in type 2 diabetes. While our selected protein panel included strong candidates for macrovascular risk prediction, few promising biomarkers emerged for microvascular outcomes, limiting predictive improvement. Several factors may underlie this difference. Biologically, microvascular beds display pronounced angiodiversity, encompassing a wide range of vessel calibers and organ-specific endothelial phenotypes adapted to distinct metabolic and physiological demands [49]. This structural and functional heterogeneity makes it less likely that a single circulating protein signature can persistently capture the full spectrum of microvascular injury. Technically, current targeted proteomic arrays appear to be optimized more toward macrovascular disease processes such as inflammation, atherosclerosis, and other cardiometabolic processes, limiting insight into microvascular pathophysiology. This may also reflect the restricted detection power of current circulating proteomic technologies to quantify low-abundance, tissue-specific proteins, as key signalling proteins in the microvasculature are often circulate at lower concentrations in the bloodstream and may fall below current detection thresholds [55]. This represents a critical research gap, as microvascular complications account for a substantial proportion of diabetes-related healthcare costs [56]. Future efforts should therefore couple expanded study sample sizes with technological advancements in proteomic profiling, including broader protein coverage and improved sensitivity, to enable a more detailed dissection of the heterogeneity underlying microvascular pathology.

Equally important is to extend these efforts to ancestrally diverse cohorts with equitable representation of underrepresented populations to improve model fairness and generalizability. Our within-ancestry comparisons were based on the premise that, although a model trained in Europeans would inevitably perform less well in non-European populations, the addition of proteomic data should at least maintain performance relative to the clinical model within each ancestry, even if the magnitude of improvement were smaller. However, our findings suggest that the predictive gains of proteomics integration depend on genetic proximity to the discovery cohort, with predictive gains diminishing or reversing as ancestry diverges. In practical terms, this implies that applying proteomics-integrated models trained primarily in European populations to patients of other ancestries could result in worse prognostic accuracy than simpler clinical-only models, despite higher cost and complexity. This pattern likely reflecting greater ancestry-related heterogeneity in molecular features than in clinical risk markers [57]. Consequently, extrapolating proteomics-based biomarkers and prediction models without accounting for ancestry-related variation could exacerbate health disparities. Although our analyses were underpowered for formal external validation, these preliminary results highlight the need for future studies in larger, ancestrally diverse cohorts to validate and extend these observations and to enhance the fairness and generalizability of proteomics-integrated prognostic models.

## Conclusions

In summary, our study lays a groundwork for further establishment of a multidisciplinary vascular complication screening framework for type 2 diabetes patients. We have identified a set of robust candidate biomarkers for future validation and clinical applications. Advancing proteomics platforms, particularly through absolute protein quantification and broader array panels, will greatly enhance the utility, reproducibility and interpretability of protein-based prediction models. Future studies should prioritize larger, ancestrally diverse cohorts, with particular efforts to improve risk prediction for microvascular complications, to enhance the clinical utility of proteomics-integrated prognosis models across vascular complications and global type 2 diabetes populations.

## Supplementary Information

Below is the link to the electronic supplementary material.

Supplementary Tables

Supplementary Figures

## Data Availability

The data that support the findings of this study were obtained from UK Biobank under application number 10205. Access to the UK Biobank data can be requested through a standard protocol (https://www.ukbiobank.ac.uk/registerapply). Cohort-level summary statistics for the data field mentioned in the manuscript can be found at https://biobank.ndph.ox.ac.uk/showcase/.

## Abbreviations

DBP: Diastolic blood pressure
EHR: Electronic health records
LOD: Limit of detection
QC: Quality control
SBP: Systolic blood pressure
T2D: Type 2 Diabetes
UKB: UK Biobank
UKB-PPP: UK Biobank Pharma Proteomics Project
