# Supplementary material for: Integration of clinical and proteomic risk factors enhances prognostic modelling of incident vascular complications in type 2 diabetes
Source: Cardiovasc Diabetol. 2026 Jan 20;25:46. doi: 10.1186/s12933-026-03083-6 (PMC12903690; doi:10.1186/s12933-026-03083-6)
Supplement: Supplementary file 2 — Supplementary Figures [file 12933_2026_3083_MOESM2_ESM.docx]

## Supplementary Figures


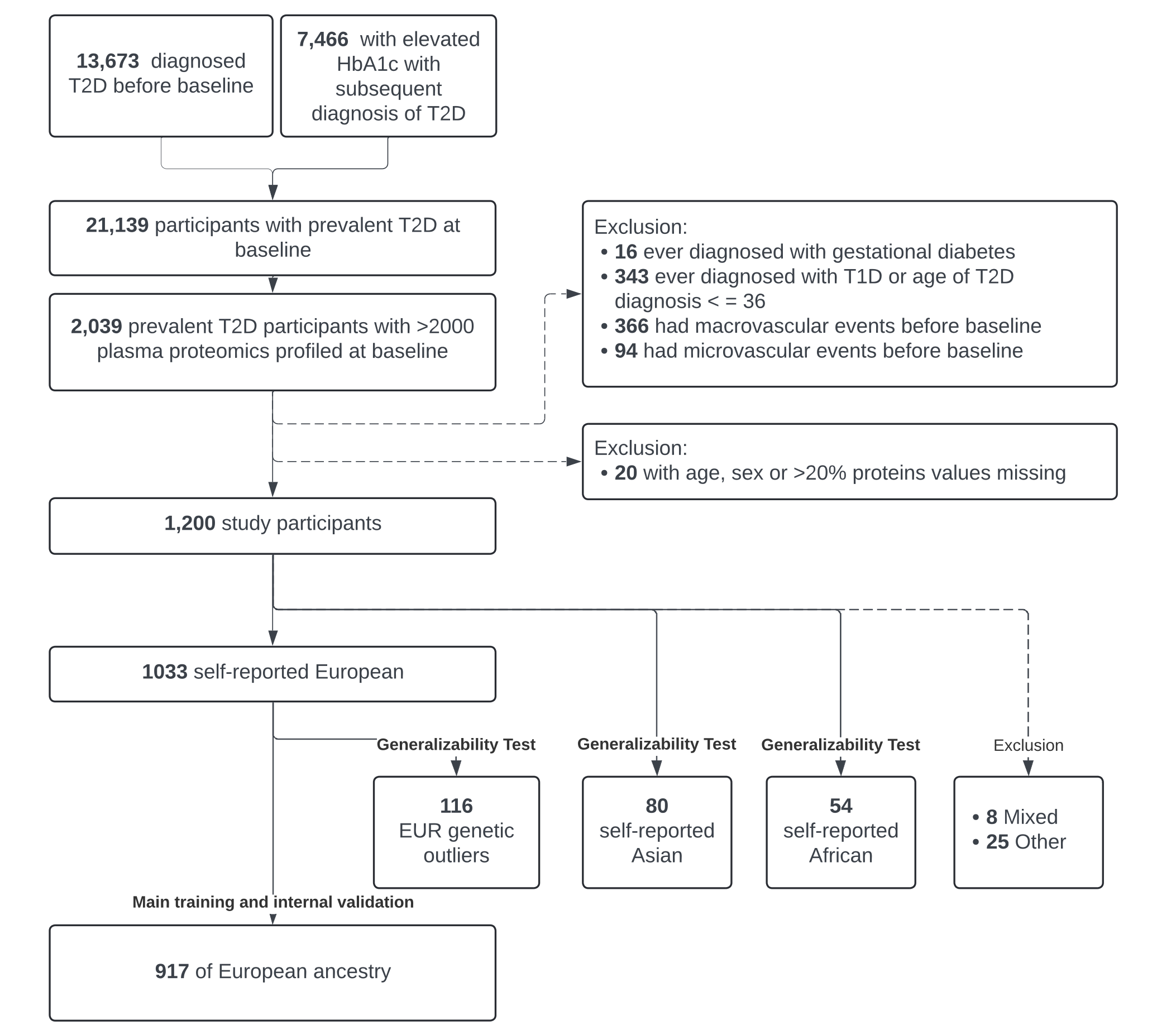


**Figure S1**. Flowchart of study participant selection within UKB. Ancestry was based on self-reporting and, for the European training set, further restricted by genetic similarity. Ancestry groups with extremely low sample sizes were excluded from analysis.


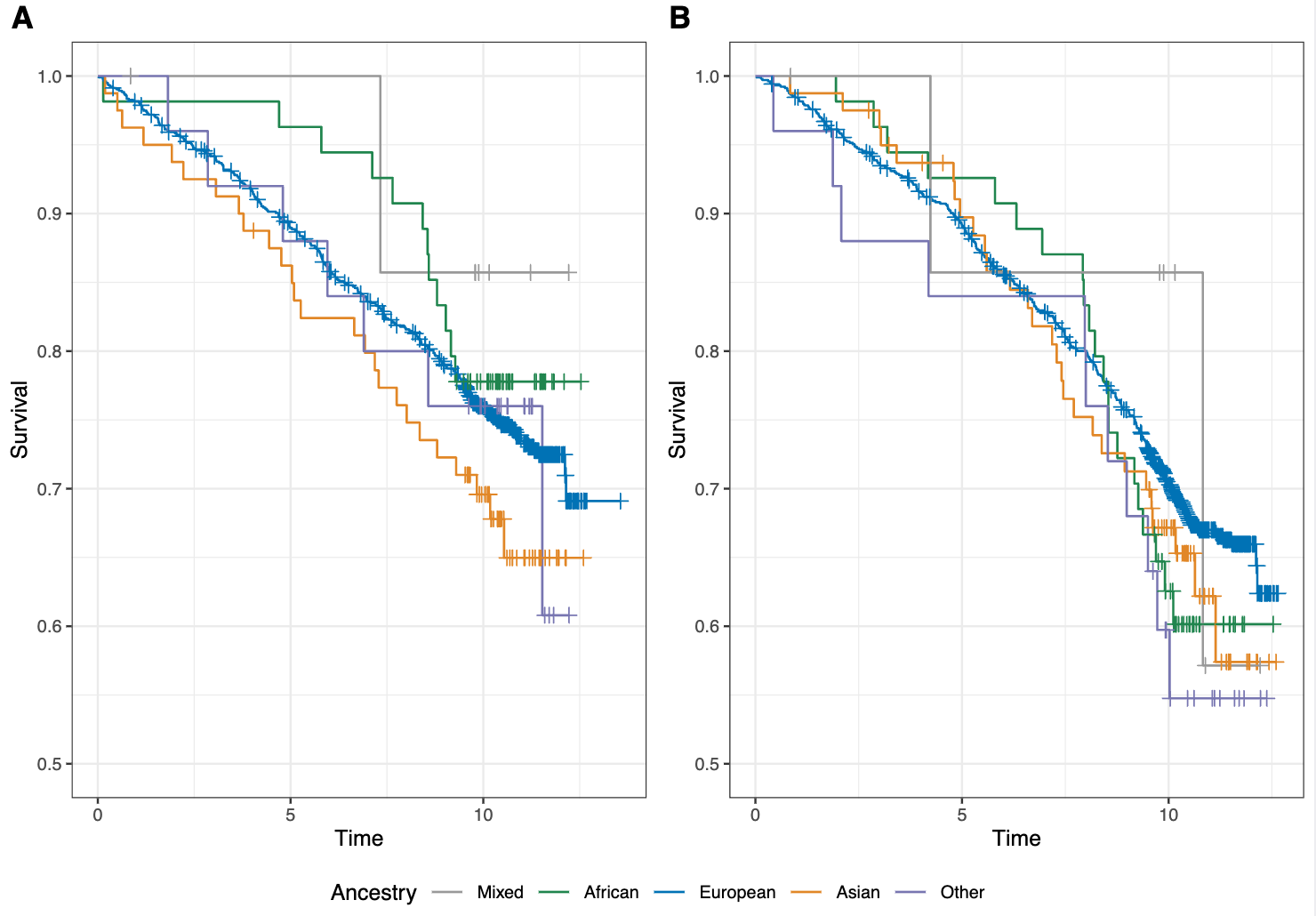


**Figure S2**. Kaplan–Meier survival curves for complication-free survival across ancestry groups within UKB. (A) Macrovascular. (B)Microvascular.


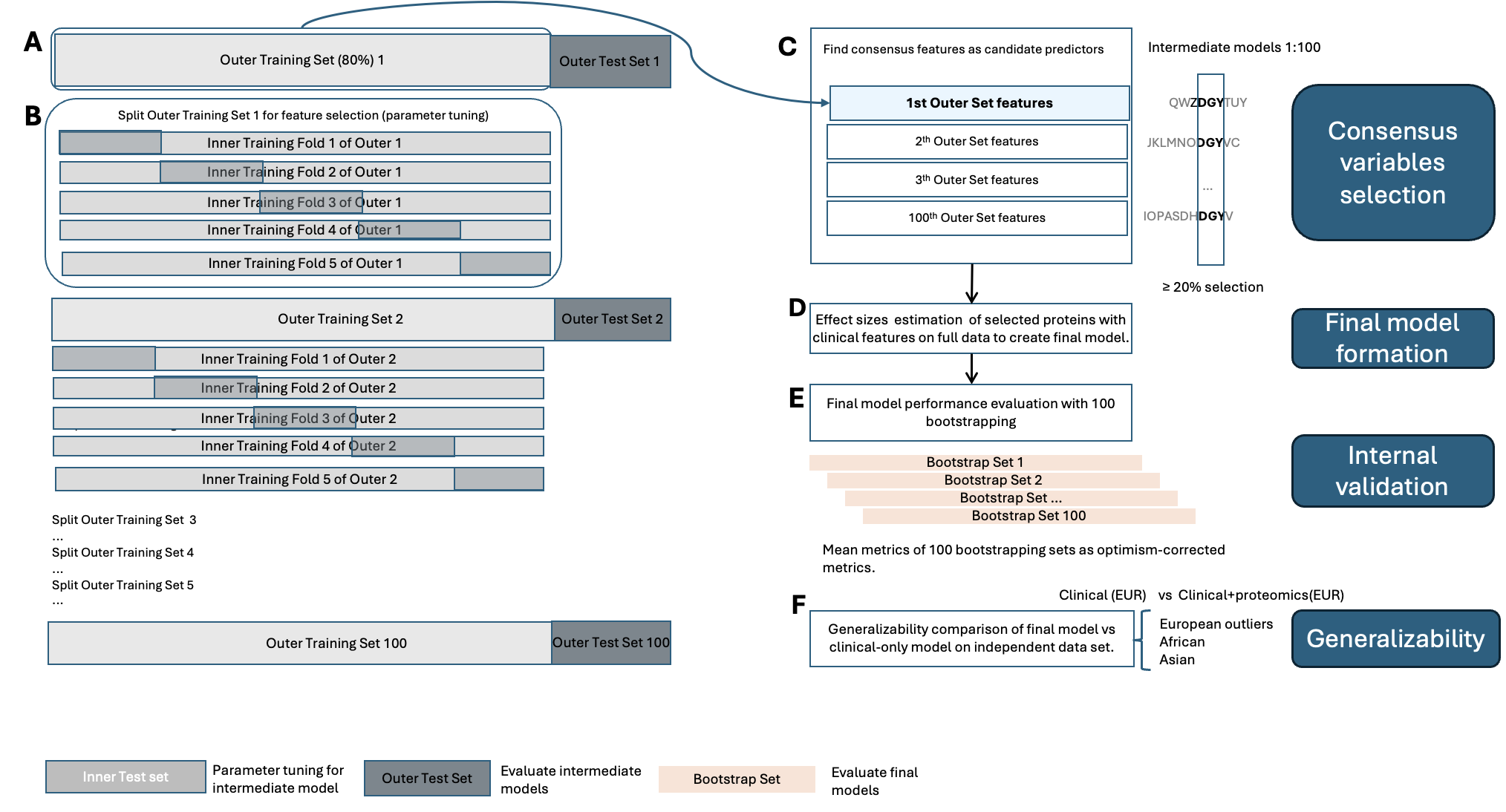


**Figure S3.** Workflow for variable selection, final model formation, internal validation, and generalizability assessment. (A) The dataset was randomly split into 80% training and 20% testing subsets, stratified by outcome status to maintain event balance. (B) Within outer training set, a five-fold inner resampling procedure was performed for hyperparameter tuning (λ) and feature selection using LASSO-Cox regression, with penalties for clinical variables fixed at zero to ensure their retention. The model achieving the highest C-index in the inner test set was selected as the intermediate model for that iteration. (C) Steps in (B) were repeated across 100 random outer splits, generating 100 intermediate models. Feature selection frequency across iterations was calculated, and proteins selected in ≥20% of models were retained as consensus variables, ensuring stability against random sampling variation. (D) All retained proteins, together with fixed clinical covariates, were used to fit the final clinical + proteomics model for each outcome on the full original dataset. A clinical-only model was also fitted for direct comparison. (E) Internal validation of the final models was performed using 100 bootstrap resamples (sampling with replacement) from the original full dataset. The average discrimination metrics across bootstrap iterations were reported as optimism-corrected performance estimates. (F) The final models were then applied to three out-of-sample datasets including European genetic outliers, Asian, and African ancestry participants within UKB. Within each ancestry, pairwise comparisons between the clinical-only and clinical + proteomics models were used to assess whether the performance gain from adding proteomics persisted beyond the training population. In addition to point estimates, ancestry-specific gains from proteomics integration (Δ = clinical + proteomics vs clinical) were also assessed using paired performance metrics across 1,000 bootstrap resamples and summarised as mean (95% CI).


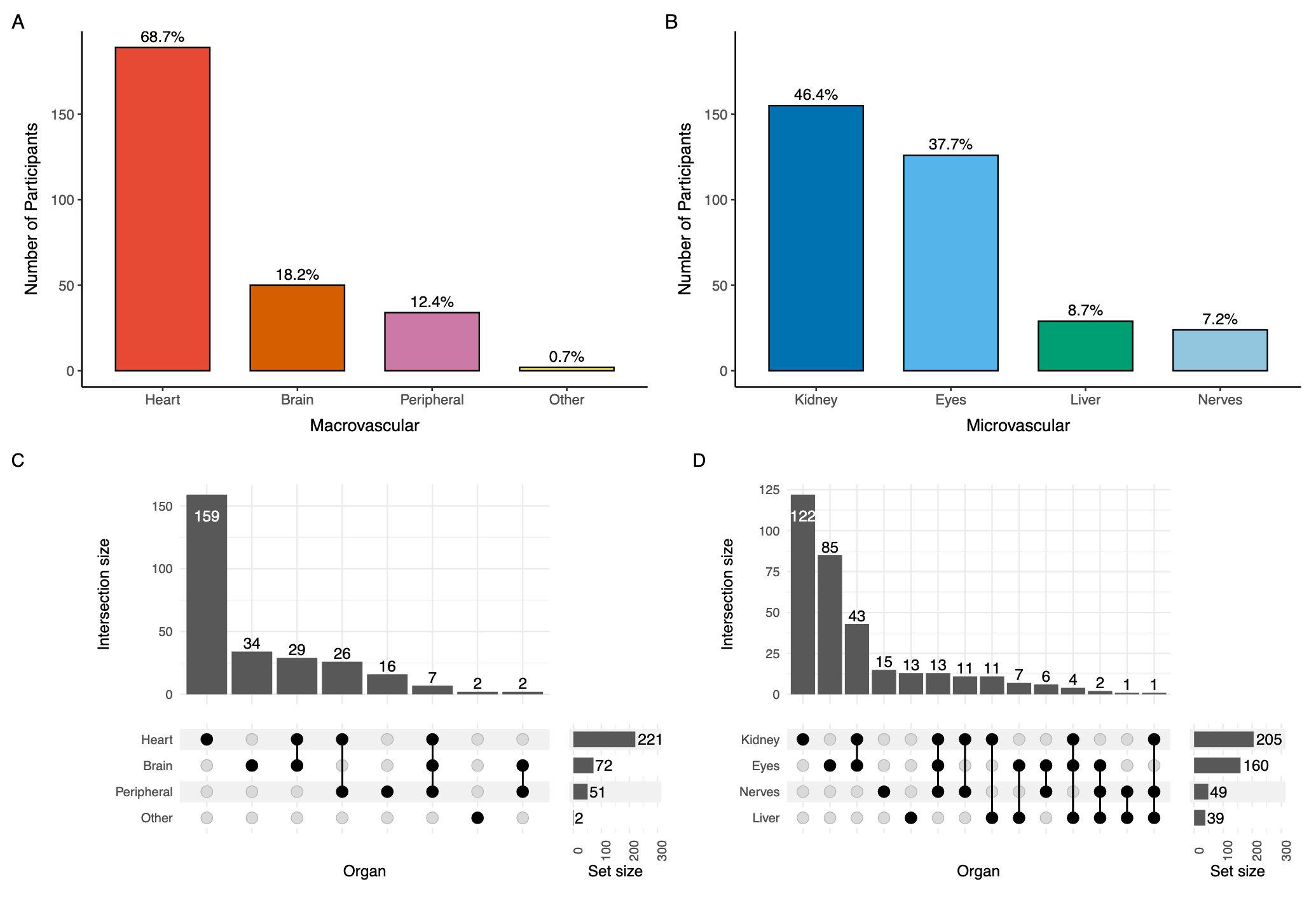


**Figure** **S4.** Distribution of first-incidence and overlap of vascular complications by organ system within each category. (A–B) Proportion of first incident complications categorized by organ system for macrovascular and microvascular outcomes, respectively. (C–D) Overlap plots illustrating the co-occurrence of individual complication types within each category.


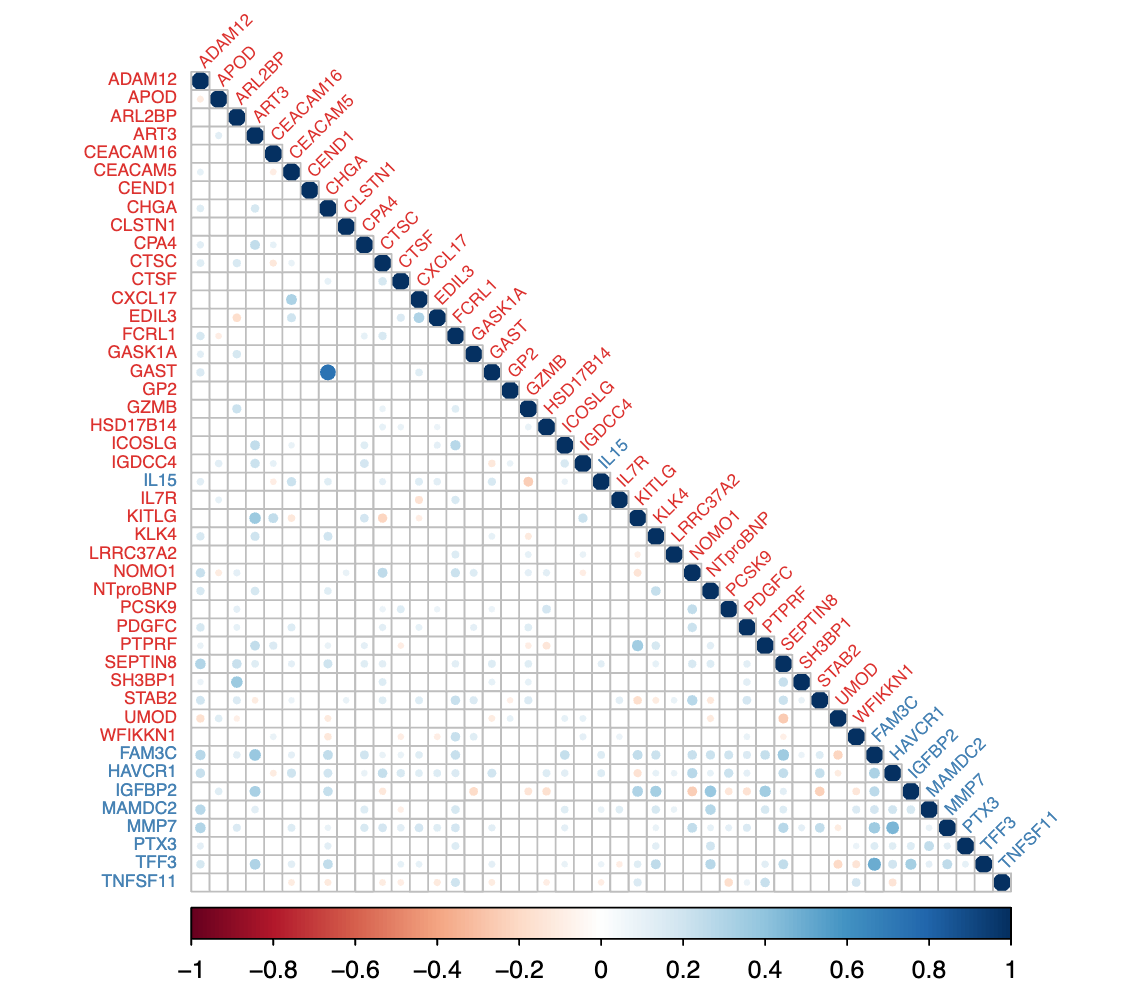


**Figure S5.** Pairwise Pearson correlation plot of selected prognostic proteins values. Labels are coloured red for proteins more frequently selected for macrovascular outcomes, and vice versa for microvascular outcomes in blue. Correlations with nominal P > 0.05 are omitted (left blank).


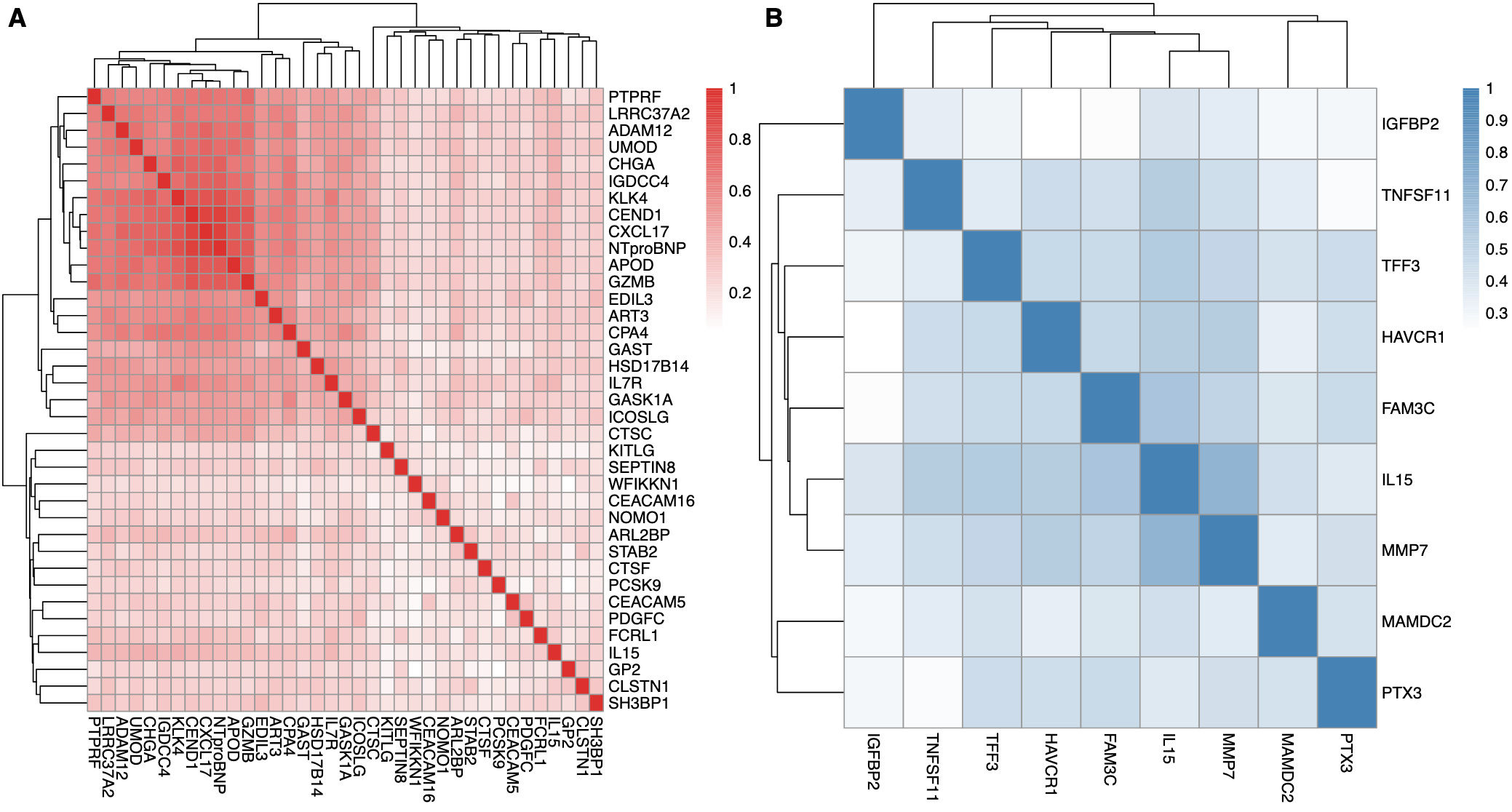


**Figure S6.** Pairwise co-selection patterns of proteins identified through repeated LASSO-Cox feature selection for macrovascular (A) and microvascular (B) models. Heatmaps show co-selection frequencies among proteins retained in the final models across 100 resampling runs, representing the probability that two proteins (i, j) were selected together (|i ∩ j| / |i ∪ j|). Rows and columns are hierarchically clustered, with higher colour intensity indicating stronger co-selection.
